# Recurrent Hypoglycemia in Diabetic Patient With Hypopituitarism: The Houssay Phenomenon

**DOI:** 10.7759/cureus.13422

**Published:** 2021-02-18

**Authors:** Mohammed Gaffar Mohammed, Javeria Baloch, Ahmed M Alsahhar, Budur M Alhabashi

**Affiliations:** 1 Internal Medicine, Dr. Sulaiman Al Habib Hospital, Riyadh, SAU; 2 Internal Medicine, College of Medicine, Alfaisal University, Riyadh, SAU; 3 Internal Medicine/General, Sulaiman Alrajhi University, Al Bukairiyah, SAU

**Keywords:** hypoglycemia, houssay phenomenon, hypopituitarism, recurrent hypoglycemia, partial empty sella turcica, short synthacten test, low cortisol, hypocotisolism

## Abstract

The association of treatment of diabetes mellitus and hypoglycemia is well described in the literature. However, the association of recurrent hypoglycemia in diabetic patients with hypopituitarism has been rarely described. This phenomenon, called Houssay phenomenon, usually occurs in individuals with a long diabetes evolution. It is caused by the failure of counter-regulatory hormones produced by the anterior pituitary gland to correct hypoglycemia. We describe this phenomenon in an elderly female known with type 2 diabetes mellitus taking insulin and oral diabetes medications. Workup showed partially empty sella on pituitary imaging. Hormonal assessment showed very low morning cortisol, low adrenocorticotropin hormone (ACTH) and zero response to synacthen. Loss of these counter-regulatory hormones leads to hypoglycemia (Houssay phenomenon). Hypopituitarism has several causes, including pituitary adenoma and traumatic brain injury as common causes among the others. In our reported case, we correlate our patient’s condition to Houssay phenomenon, for the implication of refractory hypoglycemic episodes and cortisol deficiency, all of which are the consequences of hypopituitarism. Clinicians should be aware of the link between diabetes and hypopituitarism to avoid deleterious consequences of hypoglycemia.

## Introduction

Patients with type 2 diabetes who are taking medications, such as insulin or sulfonylureas, are prone to develop hypoglycemia. However, a rare yet important cause of severe hypoglycemia is pituitary dysfunction in diabetic patients known as the Houssay phenomenon. Anterior pituitary dysfunction leads to loss of function of counter-regulatory hormones, which leads to increased insulin sensitivity causing hypoglycemia and sometimes resolution of diabetes [1]. We report a case of severe recurrent hypoglycemia in a 64-year-old female on insulin, sitagliptin, gliclazide and metformin for type 2 diabetes, secondary to hypopituitarism. She developed recurrent episodes of hypoglycemia and loss of consciousness despite discontinuation of insulin and oral hypoglycemic medication. Furthermore, brain imaging revealed a partially empty sella along with a poor Synthacten test response, low cortisol and low adrenocorticotropin hormone (ACTH) levels.

## Case presentation

A 64-year-old female was brought into the emergency department following an episode of loss of consciousness, which was associated with hypoglycemic symptoms of sweating, shivering and dizziness. The random blood sugar level on presentation to the emergency room was 40 mg/dL (2.2 mmol/L). She was given intravenous dextrose infusion to regain consciousness.

**Figure 1:**
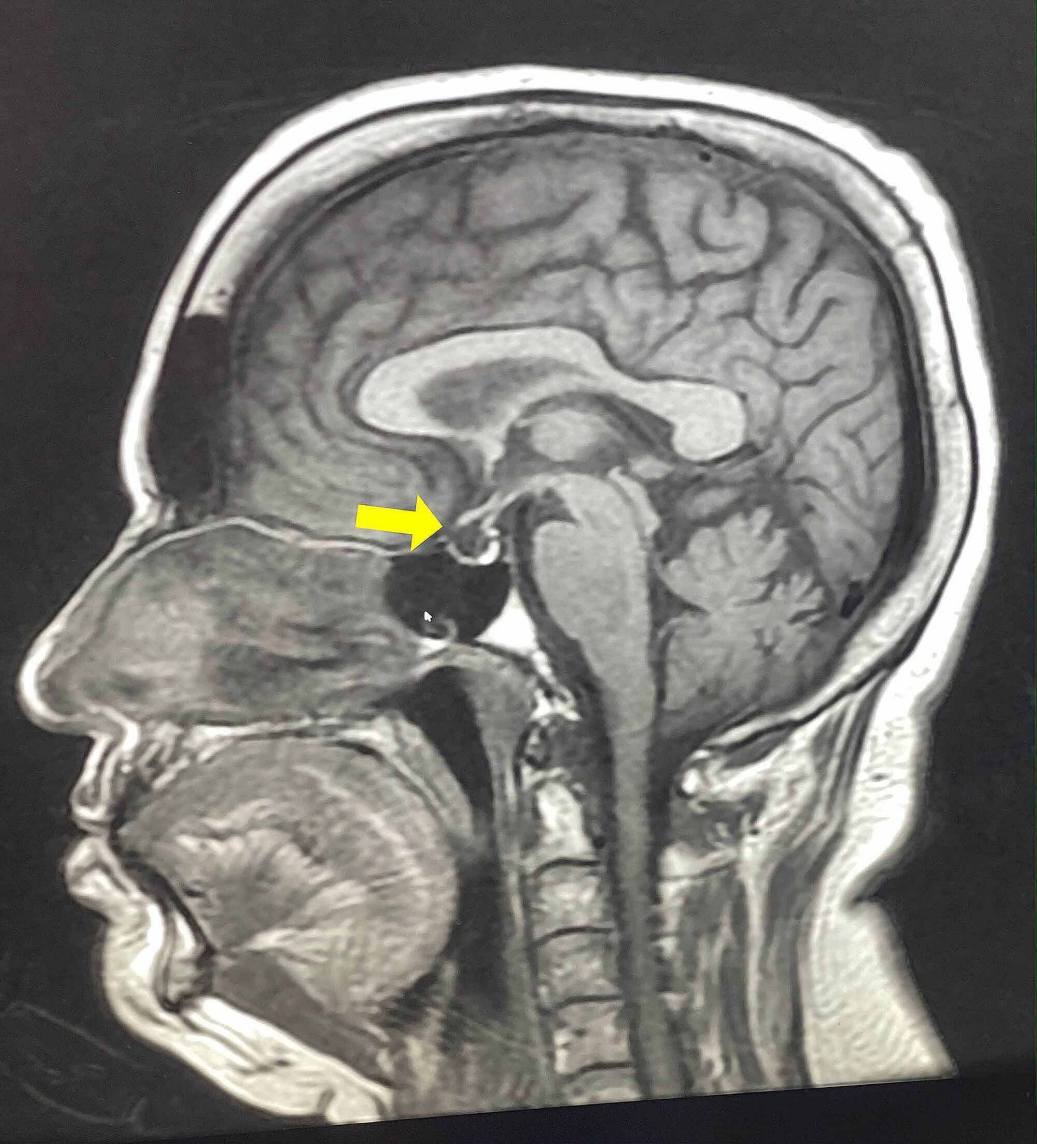
Sagittal MRI view of the hypothalamopituitary showing partially empty sella (yellow arrow)

She has diabetes for 15 years. She had been on insulin and oral diabetes medications. Her heamoglobin A1c (HbA1c) was 11.7% (104 mmol/mol) three months prior to presentation to the emergency room. However, her HbA1c was reduced to 6.5% (38 mmol/mol) upon her admission with hypoglycemia. Her blood glucose levels remained low despite discontinuation of diabetic treatment. Frequent 10% dextrose infusions were required. Nonetheless and even with close monitoring, she further developed hypoglycemic episodes requiring ICU admission. While in ICU, she developed generalized tonic-clonic seizures. Following this, a brain MRI was done, which revealed nothing significant except a partially empty sella turcica (Figure 1).

Her initial hormone workup revealed low cortisol, low follicular stimulating hormone (FSH), low luteinizing hormone (LH), low somatomedin level and low ACTH levels with normal thyroid-stimulating hormone (TSH), normal aldosterone, normal renin, normal aldosterone/renin ratio and normal prolactin. She had low morning cortisol of 25 nmol/L and low ACTH of 0.44 pmol/L. Further assessment showed poor response to short Synthacten test with cortisol levels of 24.7, 95.8 and 130.5 nmol/L at 0, 30 and 60 minutes, respectively (summarized in Table 1).

**Table 1:** Laboratory results Na = Sodium RBS = Random blood sugar TSH = Thyroid-stimulating hormone FSH = Follicular stimulating hormone LH = Luteinizing hormone ACTH = Adrenocorticotropin hormone HBA1C = Heamoglobin A1C

Test	Patient result	References
Haemoglobin (g/dL)	13.8 g/dL	11.8-14.8 g/dL
HBA1C (%)	6.4%	< 5.7%
Na (mEq/L)	126 mEq/L	136-145 mEq/L
8am serum cortisol (nmol/L)	25.60 nmol/L	101.2-535.7 nmol/L
8 pm serum cortisol (nmol/L)	8 nmol/L	79-477 nmol/L
RBS (mg)	40 mg	70-140 mg
TSH (mIU/mL)	1.29 mIU/mL	0.3-4.2 mIU/mL
Somatomedin C (IGF-1) (ng/mL)	10.6 ng/mL	42.2-169 ng/mL
Cortisol after short Synthacten test (nmol/L)	0 min: 24.7 nmol/L 30 min: 95.8 nmol/L 60 min: 130.5 nmol/L	Normal response > 420 nmol/L at 30 min
FSH (mIU/m)	4.83 mIU/m	25.8-134.8 mIU/m (Post-menopausal range)
LH (mIU/m)	2.86 mIU/m	7.7-58.5 mIU/m (Post-menopausal range)
Prolactin (mg/dL)	276 mg/dL	102-496 mg/dL
ACTH (pmol/L)	0.44 pmol/L	1.1-13.2 pmol/L
Aldosterone (ng/dL)	13.2 ng/dL	3.7-43 ng/dL
Renin (mU/mL)	29.7 mU/mL	5.3-99 mU/mL

The patient was diagnosed with primary pituitary failure with secondary adrenal insufficiency considering the investigation results. She was started on IV hydrocortisone followed by oral hydrocortisone 10 mg twice daily and was discharged on the same dose of hydrocortisone, in addition to oral hypoglycemic medications (linagliptin, empagliflozin and metformin). Since then, no more episodes of hypoglycemia have been reported and she remained stable over the remaining course of her hospital stay. Upon her follow-up visits in the outpatient clinic for the last five months, she reported improvement in her symptoms with no further hypoglycemic episodes with well-controlled self-home blood glucose monitoring.

## Discussion

Unexplained hypoglycemia in a diabetic patient should alert vigilant searches for deficiency in counter-regulatory hormones, particularly, pituitary and adrenal hormones. Development of hypoglycemia in a diabetic patient is a major health challenge to the physician and the patient owing to the fact that hypoglycemia has dangerous consequences to the patient. The occurrence of hypoglycemia in a diabetic individual can be due to different causes, including iatrogenic hypoglycemia by insulin, or oral hypoglycemic agents, in particular sulfonylurea. Moreover, diabetic patients on treatment are at risk of hypoglycemia under starvation and physical exhaustion. Other causes are renal impairment, liver disease, cardiac failure, sepsis as well as alcohol [2].

There is an array of hormones that act as counter-regulatory measures against falling blood glucose levels, particularly glucagon, adrenaline, cortisol and growth hormone. Inadequacy of these hormones may lead to hypoglycemia. One reason for inadequacy is anterior hypopituitarism, a condition in which the pituitary gland is unable to produce its hormones. The most common causes giving rise to the condition are non-functioning pituitary adenomas, traumatic brain injury and congenital defects. Other causes include hypothalamic diseases, ischemic disease and infections [3-4]. The decline of hormone production in the course of hypopituitarism is sequential; with growth hormone and gonadotropin hormones being affected first, followed by ACTH and TSH. Prolactin is rarely decreased; however, in Sheehan’s syndrome, it is substantially decreased [4]. Insufficient ACTH hormone secretion results in cortisol deficiency; therefore, hypopituitarism may cause hypoglycemic events in diabetic patients on medical treatment including insulin therapy. Furthermore, recurrent episodes of hypoglycaemia impair the glucose counter-regulatory mechanisms, predisposing to severe hypoglycaemia [5]. Notably, cortisol deficiency is associated with a lack of aldosterone like effect of cortisol on the kidneys level resulting in hyponatremia, as well as antidiuretic hormone (ADH) release is affected negatively by cortisol secretion. 

Thus, the manifestation of unexplained hyponatremia in hypoglycemic diabetic individuals suggests the possibility of hypocortisolism and hypopituitarism [6]. Interestingly, hypopituitarism is considered an uncommon cause for hypoglycemia in diabetics. However, there are a fair number of case reports representing the condition.

A phenomenon, named Houssay phenomenon, has described the possibility of recurrent hypoglycemic events in a diabetic patient due to hypopituitarism. The phenomenon was named after Bernardo Houssay, for his experiments in 1931 and his explanation of amelioration of diabetes in dogs that had undergone pancreatectomy following the loss of counter-regulatory hormones by removal of anterior pituitary gland [7]. Fewer cases reported duplication of this phenomenon in the following years after its discovery. One of the first reported cases in a human was described by Lyall and Innes in 1935 [8]. A diabetic patient who suffered from hypoglycemic events due to hypopituitarism followed by spontaneous remission to diabetes and his glucose tolerance considerably improved [8].

We correlate this phenomenon to our reported case. Our patient suffered from type 2 diabetes for 15 years. Her sugar levels were not well controlled as evident by her HbA1c done three months prior to her admission to the emergency room. Then, the patient developed recurrent episodes of hypoglycemia despite discontinuation of the insulin and administration of dextrose. Our diagnosis of Houssay phenomenon is supported by low ACTH, low basal cortisol, failure of cortisol response to synacthen and low IGF1. Classically, TSH is reduced concomitantly with ACTH in the state of hypopituitarism, but in our case, TSH appeared to be normal. Furthermore, our patient's condition improved significantly following treatment with no further reports of recurrent documented or symptomatic hypoglycemia for the last five months, which supports our decision and confirms our diagnosis further.

## Conclusions

Unlike the common causes of hypoglycemia, such as overdosing with insulin or oral hypoglycemic, starvation and increased activity, hypopituitarism is a rare but a serious cause of hypoglycemia that needs to be addressed urgently. Physicians should be conscious about the possibility of hypoglycemia in the setting of anterior pituitary dysfunction in cases presenting with repeated episodes of hypoglycemia. Moreover, the recurrent episodes may lead to even more severe episodes with time, placing the patient in a life-threatening condition. This can be avoided by early recognition of the condition and prompt delivery of the appropriate treatment.
